# Quality Assessment of Online Health Information on Thyroid Cancer in the Arabic Language: A Cross-Sectional Study

**DOI:** 10.7759/cureus.76526

**Published:** 2024-12-28

**Authors:** Alaa M Alseheimi, Saleh M Alseheimi, Kholoud A Alhysoni

**Affiliations:** 1 College of Medicine, Taibah University, Medina, SAU; 2 Department of Otolaryngology - Head and Neck Surgery, Ohud Hospital, Medina, SAU

**Keywords:** arabic, discern instrument, online health information, quality, thyroid cancer

## Abstract

Introduction

The internet is a crucial source of health information, including cancer-related topics, but the quality and reliability of these resources can vary, affecting patient decision-making.

Objectives

This study aimed to evaluate the quality of thyroid cancer-related websites in the Arabic language, using the DISCERN tool, and explore the content and sources provided by different types of websites.

Methods

A total of 78 websites were included after excluding 21 based on predefined criteria (e.g., duplicates, non-functional uniform resource locators (URLs)). The websites were categorized into commercial, non-profit, and individual types. Two independent reviewers assessed the websites using the DISCERN tool. Interrater agreement was measured using the k-score. A one-way analysis of variance (ANOVA) was used to compare DISCERN scores across website types, and Spearman’s rank correlation was used to analyze the relationship between website ranking and DISCERN scores.

Results

Almost all websites included a definition of thyroid cancer. Additionally, 15 websites (19.2%) covered the definition, clinical presentation, risk factors, diagnosis, and treatment, while 14 websites (17.9%) offered only clinical presentation, diagnosis, and treatment, and 11 websites (14.1%) offered other combinations of similar content. However, there was a lack of information regarding prognosis and predictors of outcomes following thyroid cancer surgery. The average overall DISCERN score for the 78 websites was 42.65 ± 12.35. Statistically significant differences were found in DISCERN scores across website types, with non-profit websites scoring the highest (38.93 ± 14.12), followed by commercial (37.67 ± 10.34) and individual websites (28.63 ± 10.02). A significant negative correlation was also found between website rank and DISCERN scores (Spearman’s r = -0.38, p < 0.0001).

Conclusion

The study found that non-profit websites provide higher-quality thyroid cancer information compared to commercial and individual sites. Website ranking also affects content quality, emphasizing the importance of patients assessing online resources critically. Health organizations are encouraged to improve the visibility and quality of trustworthy information.

## Introduction

Health information seeking is a way by which individuals obtain information about their health, health promotion activities, risks to health, and illnesses [1]. Over the last few decades, the internet has rapidly grown to become an important source of health information, allowing individuals to access online health information and inquire about their conditions with affordable, easy, and rapid access to a wide community [1-3].

In the United States, it is estimated that 56%-79% of individuals who use the internet seek health information [4]. Another study showed that 47.8% of South Asian adults in Canada were using the internet for seeking online health information [5]. Similar studies conducted in Saudi Arabia showed that around 68%-92% of subjects used the internet to seek online health information [6,7].

As online health information is continuously expanding, its impact has grown significantly. Accurate health information can support people's understanding and enhance patients' care and education about their health conditions, leading to better treatment choices and compliance. However, misleading and poor-quality information can be a serious source of harm to the patient [8,9]. Therefore, it is essential to assess the quality of online health information in terms of its comprehensiveness, precision, and recency.

Thyroid cancer is the most common endocrine cancer worldwide and the second most common cancer in women [10]. In recent decades, the incidence of thyroid cancer has dramatically increased in several countries [11]. According to the Global Cancer Observatory (GLOBCAN), thyroid cancer was responsible for 586,000 cancer cases worldwide in 2020, with three times the incidence in women compared to men [12]. In the United States, about 63,000 new thyroid cancer cases were expected to be diagnosed in 2014 [13], compared to 37,200 cases in 2009 [14]. In Saudi Arabia, there were around 2,833 cases of thyroid cancer in 2020 [15], with a female-to-male ratio of 1:0.3 [10].

This dramatic increase in thyroid cancer incidence has been explained by many studies as a result of increasing surveillance and diagnostic scrutiny, causing an increase in the apparent incidence of thyroid cancer [11,16-19]. However, it has also been suggested that there is a true increase in new cases of thyroid cancer owing to increased exposure to environmental factors, including ionizing radiation, alcohol, and tobacco use [17].

The thyroid cancer mortality rate has remained relatively low, with a steady decline across different countries [19], and a five-year survival rate of approximately 98.5% [20]. Nevertheless, studies have shown that patients with thyroid cancer exhibited elevated levels of distress and worry, and higher rates of anxiety and depression compared to patients with other cancers [21-24]. For this reason, cancer patients need trustworthy sources to obtain information about their disease, educate them about the available treatment options, address their concerns about treatment, and guide them in treatment decision-making [25].

Across the world, cancer patients acquire most of their disease information through the internet [21]. With the increasing number of people seeking online health information, many hospitals and healthcare providers are offering websites to share health information; nevertheless, most of these websites offer limited information [26].

Different studies have been conducted to evaluate the quality of online health information about thyroid cancer in different countries [27-30]. The scope of online health information about thyroid cancer includes the definition, diagnosis, and treatment [27], and most of these studies point to outdated websites with relatively poor-quality, incomplete, and inaccurate information regarding diagnosis and treatment [27-30]. Most of these studies, however, are based on online English health information.

Considering these facts, it is important to evaluate the online health information about thyroid cancer to guarantee accurate and updated information. To our knowledge, very few studies have been conducted to evaluate the quality of online health information about thyroid cancer, and none of them have focused on Arabic content.

The purpose of this study was to evaluate Arabic-language websites providing information about thyroid cancer, regarding its scope and accuracy.

## Materials and methods

Website selection

Our search was carried out using Google (www.google.com; Google, Inc., Mountain View, CA, USA), one of the most popular search engines in Saudi Arabia. This search was conducted on December 12, 2024, using the Arabic term 'سرطان الغدة الدرقية' ('thyroid cancer' in Arabic).

The search yielded a total of 2,790,000 results. Data was extracted from the first 10 pages, covering 99 websites. We included all websites that were written in Arabic and provided information about thyroid cancer. Websites that represented advertisements, news reports, research articles, non-readable content (including photos and videos), and those with non-functioning uniform resource locators (URLs) were excluded.

We gathered the data from the included websites and acquired the website's producer attributes from the 'About Us' page or affiliation statements. The producer attributes were arranged into three categories: non-profit organizations (e.g., public healthcare facilities, governmental organizations, academic institutions), commercial organizations (e.g., companies, private healthcare facilities), and private individuals (e.g., healthcare providers, thyroid cancer patients, and survivors).

The evaluation of the content was conducted by two reviewers (A.S. and S.S.), and any discrepancies were resolved through consensus between them.

Quality evaluation

We used the DISCERN tool for website quality evaluation. DISCERN is an instrument that was published in 1999 by Charnock et al. and has proven to be a reliable and valid tool for evaluating the quality of written health information [31].

The DISCERN questionnaire consists of 15 questions, plus an overall quality rating. These questions are categorized into three sections. Section 1 (Questions 1-8) addresses the reliability and trustworthiness of the publication. Section 2 (Questions 9-15) is concerned with details of treatment options described in the publication, and whether other possible treatment options are mentioned. Section 3 (Question 16) is an overall quality rating at the end of the instrument [32].

Each question is rated on a continuous rating scale of 1-5, where 5 is given to questions when the answer is a definite ‘yes,’ 2-4 if the publication ‘partially’ meets the criterion in the question, and 1 is given when the answer to the question is a definite ‘no.’ Question 16 is an intuitive summary of the overall quality of the publication, where 5 = High, 3 = Moderate, and 1 = Low [32].

The global DISCERN score allocated for each web page ranges from 16 to 80, with higher scores indicating better quality. An overall score less than 27 indicates ‘very poor quality,’ 27-38 indicates ‘poor quality,’ 39-50 indicates ‘fair quality,’ 51-62 indicates ‘good quality,’ and 63 and above indicates ‘excellent quality’ [9,33,34].

To ensure accuracy, two independent reviewers conducted the quality evaluation and reported the average of their overall scores.

Statistical analysis

Data analysis was performed using IBM SPSS Statistics for Windows, Version 22 (Released 2013; IBM Corp., Armonk, NY, USA). Descriptive statistics are presented as means and standard deviations. The k statistic was utilized to assess interrater agreement. To compare the mean overall DISCERN scores across the three types of websites, a one-way analysis of variance (ANOVA) was conducted. Additionally, Spearman’s rank correlation analysis was employed to evaluate the relationships between search engine rankings and overall DISCERN scores. A significance level of p < 0.05 was used to determine statistical significance.

## Results

Characteristics of included websites

**Figure 1:**
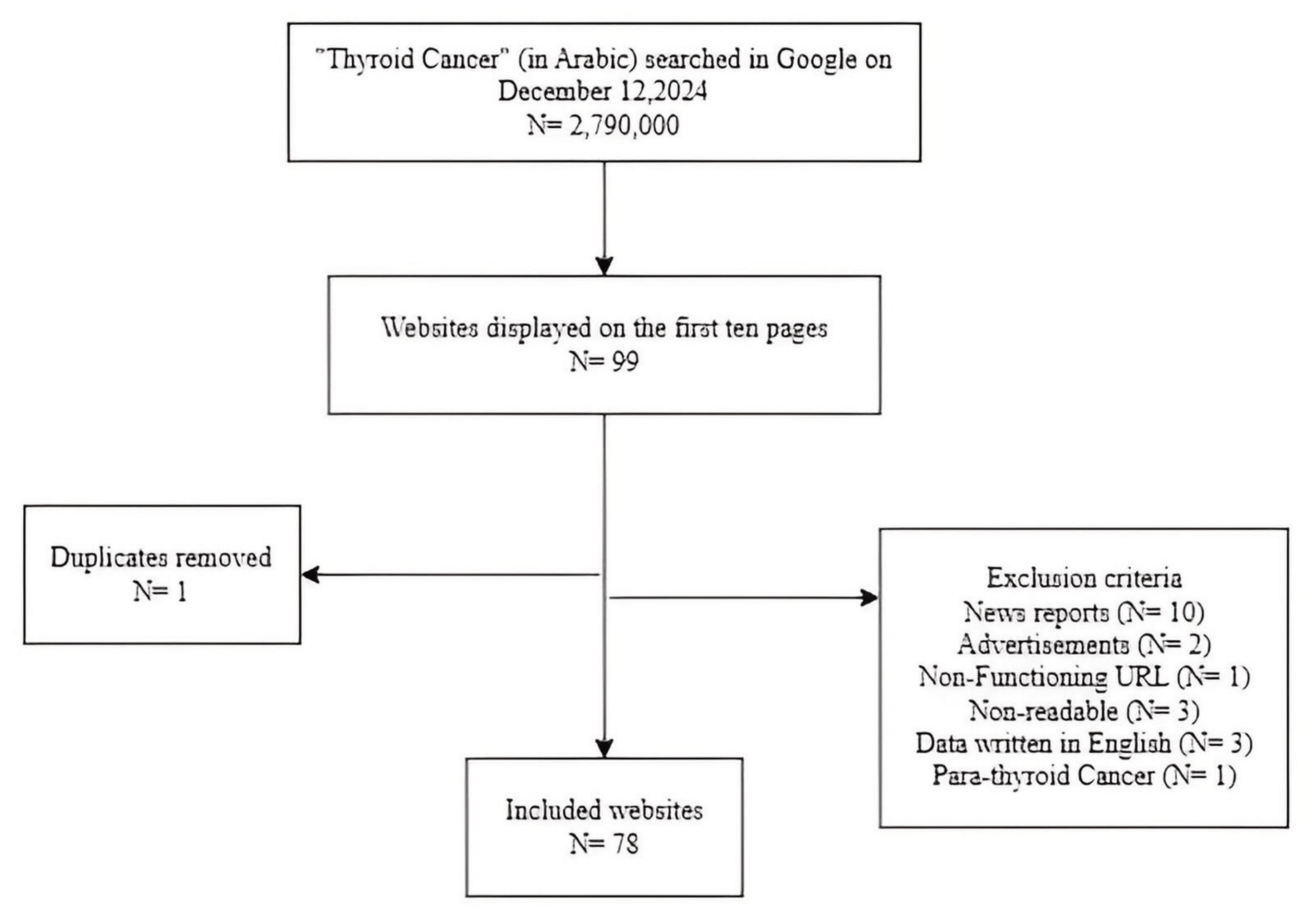
Flow diagram of the website search and selection process

A total of 78 websites were included in the study, with 21 websites excluded for the following reasons: 10 were news reports, one was a duplicate, two were advertisements, one had a non-functioning URL, three contained non-readable content, three had data written in English, and one was about parathyroid cancer (Figure 1).

Regarding the classification of website producers, 51 websites (65.4%) were commercial, 16 (20.5%) were non-profit organizations, and 11 (14.1%) were created by private individuals.

In terms of thyroid cancer-related content, almost all websites included cited the definition; 15 websites (19.2%) covered the definition, clinical presentation, risk factors, diagnosis, and treatment; 14 websites (17.9%) included a combination of clinical presentation, diagnosis, and treatment; and 11 websites (14.1%) addressed content related to the definition, clinical presentation, diagnosis, and treatment. Further details about the content of these websites can be found in Table 1. The search engine rankings of the included websites (from 1 to 78) were determined based on their order of appearance in the search results.

**Table 1:** Content items included in websites included about thyroid cancer

Content	N (%)
Definition, clinical presentation, risk factors, diagnosis, treatment	15 (19.2)
Clinical presentation, diagnosis, treatment	14 (17.9)
Definition, clinical presentation, diagnosis, treatment	11 (14.1)
Definition, clinical presentation, types and classifications, risk factors, diagnosis, treatment	9 (11.5)
Definition, epidemiology, clinical presentation, diagnosis, treatment	6 (7.7)
Definition, clinical presentation, types and classifications, diagnosis, treatment	5 (6.4)
Definition, clinical presentation, risk factors, treatment	5 (6.4)
Staging, clinical presentation, risk factors, diagnosis, treatment	5 (6.4)
Special patient groups (children), clinical presentation, diagnosis, treatment, follow-up	4 (5.1)
Types and classifications, clinical presentation, diagnosis, treatment	4 (5.1)

Quality evaluation using DISCERN

Table 2 presents the average DISCERN scores for all 78 websites, as evaluated by two reviewers, across the 16 DISCERN items and the overall scores. The k-score was used to assess interrater agreement for each item, with a cutoff of k > 0.40 indicating an acceptable level of agreement. All items met this threshold, demonstrating moderate and acceptable reliability of the DISCERN system. The mean scores for the 16 items varied considerably, ranging from 1.13 ± 0.67 to 4.45 ± 1.35. The overall mean DISCERN score for the 78 websites was 42.65 ± 12.35.

**Table 2:** Average scores for each of the 16 DISCERN items *SD: Standard deviation; **k: Kappa statistics

DISCERN item	Average scores (SD*)	Interrater agreement (k**)
1. Provides clear aims	2.10 (1.66)	0.65
2. Achieves its aims	2.77 (0.65)	0.64
3. Provides relevant information	3.97 (1.29)	0.70
4. Provides sources of information	1.55 (1.08)	0.45
5. Provides information production date	1.85 (1.26)	0.50
6. Balanced and unbiased	4.45 (1.35)	0.45
7. Provides additional sources of information	1.62 (1.45)	0.55
8. Refers to areas of uncertainty	3.26 (1.47)	0.68
9. Describes how each treatment works	1.68 (1.27)	0.75
10. Describes the benefits of each treatment	3.13 (1.78)	0.60
11. Describes the risks of each treatment	1.62 (1.21)	0.45
12. Describes what would happen if no treatment is used	1.13 (0.67)	0.55
13. Describes how treatment affects quality of life	1.31 (0.86)	0.50
14. Makes clear that there may be more than one possible treatment choice	2.79 (1.81)	0.59
15. Supports shared decision-making	1.56 (1.40)	0.75
16. Overall quality	2.73 (1.02)	0.60
Overall scores	42.65 (12.35)	-

Table 3 compares the mean overall DISCERN scores across three types of websites. In the one-way ANOVA analysis, the non-profit group had significantly higher overall mean scores (38.93 ± 14.12) compared to both the commercial group (37.67 ± 10.34) and the individual group (28.63 ± 10.02), with a p-value of 0.04.

**Table 3:** Comparison of mean overall DISCERN scores across the three types of websites *: Statistically significant; ANOVA: Analysis of variance

Categorization of websites included	N (%)	Mean overall scores	F test (ANOVA)	p-value
Commercial	51 (65.4)	37.67 ± 10.34	3.15	0.04*
Non-profit	16 (20.5)	38.93 ± 14.12		
Individuals	11 (14.1)	28.63 ± 10.02		

Correlation between search engine ranking and overall DISCERN scores

**Figure 2:**
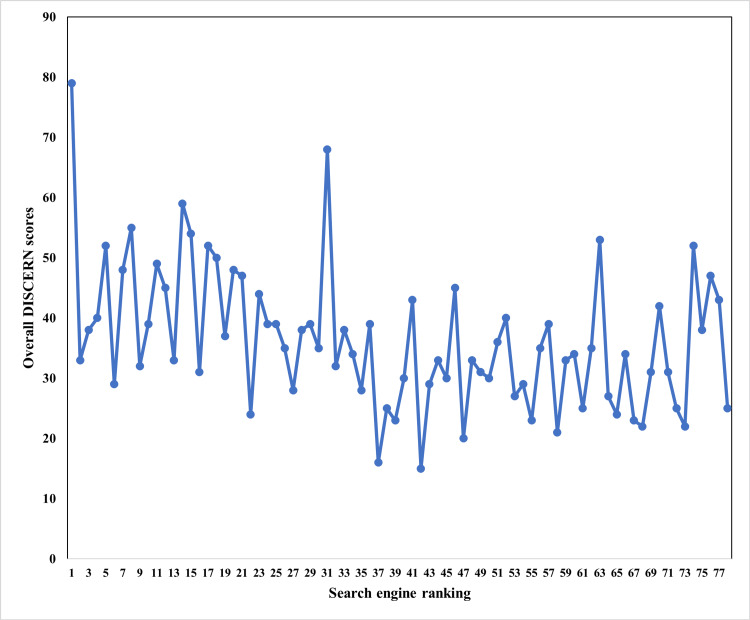
Correlations between search engine ranking and website quality and overall DISCERN scores

Figure 2 illustrates the correlations between search engine ranking and overall DISCERN scores. Spearman's rank correlation analysis revealed a significant negative correlation between website rank and overall DISCERN scores (Spearman’s r = -0.38, p < 0.0001). Subgroup analysis by website producers revealed a significantly negative correlation for commercial websites (r = -0.39, p = 0.004), and a negative correlation for non-profit websites (r = -0.42, p = 0.10). For individual websites, the correlation was not statistically significant, but positive (r = 0.39, p = 0.22).

## Discussion

Thyroid cancer information is widely available online, but its quality varies significantly. Most websites provided comprehensive coverage of diagnosis and treatment, along with relevant risk factors and staging, while some also addressed special patient groups and follow-up care [27-30]. Several studies have used the DISCERN instrument to analyze Chinese-language websites related to thyroid cancer [35] and diseases other than thyroid cancer [36-38]. The findings revealed that almost all websites included a definition of thyroid cancer. Additionally, 15 websites (19.2%) covered a combination of definition, clinical presentation, risk factors, diagnosis, and treatment, while 14 websites (17.9%) provided only clinical presentation, diagnosis, and treatment, and 11 websites (14.1%) offered another combination of similar content. In 2019, a study on 100 websites found that 26% mentioned authorship and 56% cited sources. Commonly, websites addressed the definition (94%), diagnosis (92%), and treatment (94%) of thyroid cancer [35]. Unlike our study findings, diagnosis and treatment information was often incomplete or inaccurate; diagnosis information was complete and accurate in only 50% of cases, and treatment in 47%. Additionally, only 2% of websites were comprehensible to those without a high school education. Among 83 websites contacted with patient questions, 50 responded, with 48 providing replies within one week. The key difference between our study and that study lies in the accuracy and completeness of information [27]. The 2019 study found that diagnosis and treatment information on websites was often incomplete or inaccurate, with only 50% of websites offering accurate diagnoses and 47% providing accurate treatment information. In contrast, our study suggests that the information on these topics was better organized, potentially indicating improved quality or completeness. While the 2019 study evaluated website responsiveness, finding that 60% replied to inquiries within a week, our study did not assess this aspect, highlighting a gap in interactive features. Additionally, our study used the DISCERN instrument for quality assessment, which was not used in the previous study.

A recent study assessed online health information for head and neck cancer (HNC) by reviewing 32 websites. The study found that the websites offered limited information on treatment, advice, support during treatment, and strategies for adjusting to life with and beyond HNC. However, this study did not evaluate the quality of the websites, as they included patient narratives, and most web-based interventions were text-based [39]. Also, a quality analysis of 60 thyroid cancer treatment websites, referencing the 2015 American Thyroid Association (ATA) guidelines, used four validated measures: DISCERN, JAMA benchmark criteria, HONcode certification, and the SAM method. The analysis revealed that none of the websites included all updates from the ATA guidelines, and only 18.2% referenced them. Furthermore, 31.8% covered all three treatment options, and 28.2% addressed 29 essential decision-making items. Overall, DISCERN scores were 'fair,' with 29.9% meeting JAMA benchmarks and 40.9% being HONcode certified [35].

The previous studies evaluating online information on thyroid cancer employed measurement instruments different from the one used in our study. For instance, Kuenzel et al. [28] utilized a standardized tool from the German Cancer Society to assess the quality of thyroid cancer patient information, revealing significant variation depending on the website provider. Most providers were media outlets and health organizations, offering information of relatively poor quality. The majority of websites also provided low-quality content overall. Another study used a Delphi panel of endocrine experts and practice guidelines to evaluate the top 50 thyroid cancer websites [29]. The study found that most websites were not solely focused on thyroid cancer (72%), included advertisements (72%), lacked references (66%), and were privately sponsored (50%). Only 38% had been updated in the past two years. 'Government' and 'Non-Profit' websites were the most consumer-friendly. The mean quality score for medical content was 38%, with the highest score in 'Anatomy/Physiology' (55%) and the lowest in 'Surgery' (29%). The low-quality score was mainly due to information deficiencies, rather than inaccuracies.

A similar study on breast cancer reported comparable findings, particularly regarding the mean DISCERN scores and the analysis by website type [9]. The mean score in that study was 50.27 ± 4.14, with non-profit websites scoring the highest and private individual websites the lowest. In contrast, our study found an overall mean score of 42.65 ± 12.35, with non-profit websites scoring significantly higher (38.93 ± 14.12). Unlike the breast cancer study, which found no significant correlation between search engine ranking and website quality, our study demonstrated a significant negative correlation between website rank and DISCERN scores (r = -0.38, p < 0.0001). Subgroup analysis revealed a negative correlation for both commercial and non-profit websites and a non-significant positive correlation for individual websites. This discrepancy may be due to the use of the Baidu search engine in the breast cancer study, which analyzed a smaller sample of websites (n = 49) and may yield different results compared to the larger sample (n = 78) in our study, which used the popular Google search engine. Consistent with our study results, Udayanga et al. [40] reported a total DISCERN score of 50% for the 49 websites included in their analysis, with 14 websites (28.5%) scoring lower. They also found a significant negative correlation between website ranking and DISCERN scores (p = 0.001).

Finally, consistent with findings from other studies that assessed the quality of online information about thyroid cancer [27,28,40] and other cancers [9,33,39], this study also found a lack of information regarding prognosis and post-operative care following thyroid cancer surgery, including outcome predictors. This finding suggests that, despite efforts to assess and improve online cancer information, critical aspects like post-surgical care remain underrepresented.

This study has several strengths. First, it utilized the DISCERN instrument, a reliable and validated tool, to assess the quality of thyroid cancer-related information across various websites. This ensured a thorough and systematic evaluation of the content. Additionally, the inclusion of a diverse range of website types - commercial, non-profit, and individual - allowed for a comprehensive comparison of how the source of information influences content quality. The large sample size of 78 websites provided robust data, enhancing the generalizability of the findings. Moreover, the study’s detailed subgroup and correlation analyses, particularly regarding website rank, contributed valuable insights into factors influencing information quality. To the best of our knowledge, this study is the first to assess the quality of online health information about thyroid cancer in Arabic content.

However, there are also limitations to consider. The reliance on search engine rankings to select websites could have introduced bias, as higher-ranking websites may not always be the most relevant or accurate. Additionally, the exclusion of certain websites for reasons such as duplicate content or non-functioning URLs may have led to a potential loss of valuable information and introduced selection bias. Although the DISCERN tool is standardized, the assessment still involved subjective judgments, and slight interrater variability could have impacted the consistency of evaluations. Finally, the cross-sectional nature of the study means that it only provides a snapshot of online content quality at a specific point in time, without accounting for changes over time in the quality of available information.

## Conclusions

In conclusion, this study highlights the significant variation in the quality of thyroid cancer-related information available on the internet. Our findings indicate that non-profit websites tend to offer higher-quality content compared to commercial and individual websites, as measured by the DISCERN instrument. This underscores the importance of source credibility when seeking health information online. Moreover, the study reveals that website ranking plays a crucial role in determining content quality. Higher-ranked websites were generally associated with more reliable information, suggesting that search engine algorithms may inadvertently prioritize certain types of content over others. This presents an opportunity for health organizations and trusted entities to improve the visibility of reliable, evidence-based information, ensuring that patients have easy access to accurate resources.

Future research should explore ways to enhance the quality and accessibility of online health information, as well as strategies for improving public awareness of trustworthy sources. Health organizations and medical professionals can play a vital role in guiding individuals to reliable resources, ultimately supporting better-informed decision-making and patient empowerment.
